# *catena*-Poly[[diacetonitrile­copper(I)]-μ-dicyanamido]

**DOI:** 10.1107/S1600536811047052

**Published:** 2011-11-19

**Authors:** Benjamin Oelkers, Marion Stricker, Jörg Sundermeyer

**Affiliations:** a Philipps-Universität Marburg, Fachbereich Chemie, Hans-Meerwein-Strasse, 35032 Marburg, Germany

## Abstract

The crystal structure of the title compound, [Cu(C_2_N_3_)(C_2_H_3_N)_2_]_*n*_, features zigzag chains along the *a* axis that consist of alternating [Cu(MeCN)_2_] and dicyanamide units, the latter acting as bidentate ligands *via* both terminal N atoms. The Cu atom shows a slightly distorted tetra­hedral coordination sphere. The anionic and neutral ligands lie on different mirror planes (perpendicular to the *b* and *a* axis, respectively), while the Cu atom is situated on their inter­section. The asymmetric unit comprises one fourth of the formula unit.

## Related literature

For ionic liquids (ILs) with dicyanamide anions, see: MacFarlane *et al.* (2001 ▶). For copper-based ILs, see: Stricker *et al.* (2010 ▶). For solvent-free [Cu(dicyanamide)] and its monoadduct with acetonitrile, see: Bessler *et al.* (2000 ▶); Batten *et al.* (2000 ▶).
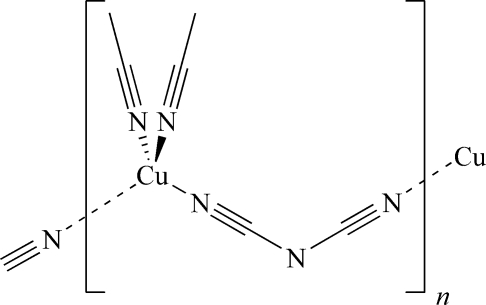


## Experimental

### 

#### Crystal data

[Cu(C_2_N_3_)(C_2_H_3_N)_2_]
*M* *_r_* = 211.71
Orthorhombic, 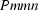

*a* = 7.5222 (5) Å
*b* = 10.5307 (11) Å
*c* = 5.5378 (4) Å
*V* = 438.67 (6) Å^3^
*Z* = 2
Mo *K*α radiation
μ = 2.44 mm^−1^
*T* = 100 K
0.45 × 0.27 × 0.12 mm

#### Data collection

Stoe IPDS 2 diffractometer
Absorption correction: multi-scan (Blessing, 1995 ▶) *T*_min_ = 0.375, *T*_max_ = 0.783
4132 measured reflections
528 independent reflections
510 reflections with *I* > 2σ(*I*)
*R*_int_ = 0.097

#### Refinement

*R*[*F* ^2^ > 2σ(*F* ^2^)] = 0.032
*wR*(*F* ^2^) = 0.066
*S* = 1.13
528 reflections
39 parameters
H-atom parameters constrained
Δρ_max_ = 0.89 e Å^−3^
Δρ_min_ = −1.05 e Å^−3^

### 

Data collection: *X-AREA* (Stoe & Cie, 2001 ▶); cell refinement: *X-AREA*; data reduction: *X-AREA*; program(s) used to solve structure: *SIR92* (Altomare *et al.*, 1993 ▶); program(s) used to refine structure: *SHELXL97* (Sheldrick, 2008 ▶); molecular graphics: *DIAMOND* (Brandenburg, 2007 ▶); software used to prepare material for publication: *WinGX* (Farrugia, 1999 ▶).

## Supplementary Material

Crystal structure: contains datablock(s) I, global. DOI: 10.1107/S1600536811047052/hp2020sup1.cif

Structure factors: contains datablock(s) I. DOI: 10.1107/S1600536811047052/hp2020Isup2.hkl

Additional supplementary materials:  crystallographic information; 3D view; checkCIF report

## Figures and Tables

**Table:** 

Cu1—N2	1.974 (2)
Cu1—N3	2.021 (2)
N1—C1	1.312 (3)
N2—C1	1.155 (3)

**Table:** 

N2^i^—Cu1—N2	110.34 (11)
N2—Cu1—N3	111.79 (4)
N3^i^—Cu1—N3	98.91 (11)
C1^ii^—N1—C1	117.0 (3)
C1—N2—Cu1	172.85 (18)
N2—C1—N1	176.2 (2)

Symmetry codes: (i) 
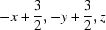
; (ii) 
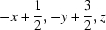
.

**Fig. 1.**
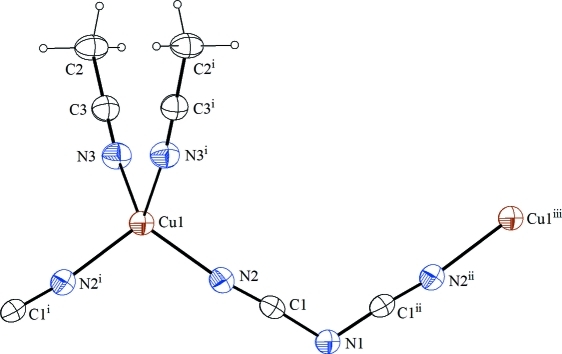
Molecular structure of the title compound. Displacement ellipsoids are shown for 50% probability. Symmetry codes: (i) 3/2–x, 3/2–y, z; (ii) 1/2–x, 3/2–y, z; (iii) -1 + x, y, x.

**Fig. 2.**
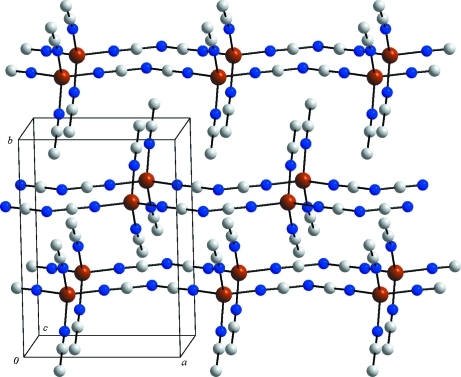
Packing diagram of the title compound, view along [1 1 10].

## supplementary crystallographic information

### Comment

Salts with dicyanamide (DCA) as anion have gained some interest recently because of their use as ionic liquids (ILs) with low viscosities (MacFarlane *et al.*, 2001). During our ongoing investigations on copper-containing ILs (Stricker *et al.*, 2010), we found that upon heating a mixture of Cu(DCA) and [BMIM](DCA) ([BMIM]^+^ = 1-*n*-butyl-3-methylimidazolium) in acetonitrile the title compound [Cu(DCA)(MeCN)_2_]_∞_ is formed instead of the expected product [BMIM][Cu(DCA)_2_]. It is interesting to note that slightly different conditions ([Cu(MeCN)_4_]ClO_4_, (Ph_3_P=N=PPh_3_)(DCA), acetonitrile/acetone, ambient temperature) lead to the monoadduct [Cu(DCA)(MeCN)]_∞_ (Batten *et al.*, 2000), while solvent-free copper(I)-dicyanamide results from reduction of an aqueous solution of Na(DCA) and CuSO_4_ with NaHSO_3_ (Bessler *et al.*, 2000).

The crystal structure of the title compound features zigzag chains along the *a* axis that consist of alternating [Cu(MeCN)_2_] and dicyanamide units. The latter act as bidentate ligands *via* both terminal nitrogen atoms, the copper atoms show a slightly distorted tetrahedral coordination sphere (Fig. 1). The anionic and neutral ligands lie on different mirror planes (perpendicular to the *b* and *a* axis, respectively) while the metal is situated on their intersection. The asymmetric unit comprises one fourth of the formula unit.

The comparison of the title compound [Cu(DCA)(MeCN)_2_]_∞_ and the monoadduct [Cu(DCA)(MeCN)]_∞_ (Batten *et al.*, 2000) shows similarities in terms of bond lengths and angles. In fact, both structures can locally be related to each other by formally breaking the copper-imide bonds of the latter and replacing them with acetonitrile ligands. The obtained structural motif is then very similar to the one-dimensional polymer building up the title compound (Fig. 2). Thus a partial depolymerization of [Cu(DCA)(MeCN)]_∞_ formally leads to the reported structure of [Cu(DCA)(MeCN)_2_]_∞_ without additional bond breaking.

### Experimental

To a mixture of Cu(DCA) (68 mg, 0.52 mmol) and [BMIM](DCA) (108 mg, 0.53 mmol; [BMIM]^+^ = 1-*n*-butyl-3-methylimidazolium) acetonitrile (60 ml) was added. Although the mixture was heated to 80 °C for 4.5 h, some amorphous solid remained. Upon slow cooling to room temperature colourless crystals of the title compound formed.

### Refinement

Hydrogen atoms of the methyl groups were placed on idealized positions and refined using a riding model with *U*_iso_(H) = 1.5 × *U*_eq_(C) and C–H bond lengths of 0.98 Å.

### Crystal data

**Table:**

[Cu(C_2_N_3_)(C_2_H_3_N)_2_]	*F*(000) = 212
*M**_r_* = 211.71	*D*_x_ = 1.603 Mg m^−^^3^
Orthorhombic, *P**m**m**n*	Mo *K*α radiation, λ = 0.71073 Å
Hall symbol: -P 2ab 2a	Cell parameters from 9024 reflections
*a* = 7.5222 (5) Å	θ = 1.9–27.1°
*b* = 10.5307 (11) Å	µ = 2.44 mm^−^^1^
*c* = 5.5378 (4) Å	*T* = 100 K
*V* = 438.67 (6) Å^3^	Prism, colourless
*Z* = 2	0.45 × 0.27 × 0.12 mm

### Data collection

**Table:**

Stoe IPDS 2 diffractometer	528 independent reflections
Radiation source: fine-focus sealed tube	510 reflections with *I* > 2σ(*I*)
graphite	*R*_int_ = 0.097
Detector resolution: 6.67 pixels mm^-1^	θ_max_ = 26.7°, θ_min_ = 3.3°
rotation method scans	*h* = −8→9
Absorption correction: multi-scan (Blessing, 1995)	*k* = −13→13
*T*_min_ = 0.375, *T*_max_ = 0.783	*l* = −7→6
4132 measured reflections	

### Refinement

**Table:**

Refinement on *F*^2^	Primary atom site location: structure-invariant direct methods
Least-squares matrix: full	Secondary atom site location: difference Fourier map
*R*[*F*^2^ > 2σ(*F*^2^)] = 0.032	Hydrogen site location: inferred from neighbouring sites
*wR*(*F*^2^) = 0.066	H-atom parameters constrained
*S* = 1.13	*w* = 1/[σ^2^(*F*_o_^2^) + (0.0425*P*)^2^ + 0.0839*P*] where *P* = (*F*_o_^2^ + 2*F*_c_^2^)/3
528 reflections	(Δ/σ)_max_ < 0.001
39 parameters	Δρ_max_ = 0.89 e Å^−^^3^
0 restraints	Δρ_min_ = −1.05 e Å^−^^3^

### Special details

**Table:**

Geometry. All s.u.'s (except the s.u. in the dihedral angle between two l.s. planes) are estimated using the full covariance matrix. The cell s.u.'s are taken into account individually in the estimation of s.u.'s in distances, angles and torsion angles; correlations between s.u.'s in cell parameters are only used when they are defined by crystal symmetry. An approximate (isotropic) treatment of cell s.u.'s is used for estimating s.u.'s involving l.s. planes.
Refinement. Refinement of *F*^2^ against ALL reflections. The weighted *R*-factor *wR* and goodness of fit *S* are based on *F*^2^, conventional *R*-factors *R* are based on *F*, with *F* set to zero for negative *F*^2^. The threshold expression of *F*^2^ > 2σ(*F*^2^) is used only for calculating *R*-factors(gt) *etc*. and is not relevant to the choice of reflections for refinement. *R*-factors based on *F*^2^ are statistically about twice as large as those based on *F*, and *R*- factors based on ALL data will be even larger.The methyl group was modelled using AFIX 133 as a riding group with idealized geometry. As the most favourable conformation was found to be symmetric with respect to a mirror plane, two protons with half occupancy were generated at symmetry-related positions. These two symmetry-dependent protons were combined by eliminating one of them, setting the other one to full occupancy and finally changing the AFIX code to 03 in order to retain the idealized geometry.

### Fractional atomic coordinates and isotropic or equivalent isotropic displacement parameters (Å^2^)

**Table:**

	*x*	*y*	*z*	*U*_iso_*/*U*_eq_	
Cu1	0.7500	0.7500	0.37747 (6)	0.02393 (18)	
N1	0.2500	0.7500	0.8017 (5)	0.0267 (5)	
N2	0.5346 (3)	0.7500	0.5810 (3)	0.0270 (4)	
N3	0.7500	0.8958 (2)	0.1402 (3)	0.0299 (4)	
C1	0.3987 (3)	0.7500	0.6779 (4)	0.0226 (4)	
C2	0.7500	1.0397 (2)	−0.2413 (4)	0.0318 (5)	
H2A	0.7500	0.9863	−0.3861	0.048*	
H2B	0.8562	1.0937	−0.2407	0.048*	
C3	0.7500	0.95947 (19)	−0.0270 (4)	0.0267 (4)	

### Atomic displacement parameters (Å^2^)

**Table:**

	*U*^11^	*U*^22^	*U*^33^	*U*^12^	*U*^13^	*U*^23^
Cu1	0.0242 (3)	0.0252 (2)	0.0224 (3)	0.000	0.000	0.000
N1	0.0233 (12)	0.0342 (13)	0.0224 (12)	0.000	0.000	0.000
N2	0.0241 (9)	0.0312 (9)	0.0256 (8)	0.000	−0.0021 (7)	0.000
N3	0.0348 (11)	0.0268 (10)	0.0281 (11)	0.000	0.000	0.0007 (7)
C1	0.0258 (10)	0.0214 (8)	0.0205 (9)	0.000	−0.0044 (8)	0.000
C2	0.0463 (13)	0.0246 (10)	0.0245 (11)	0.000	0.000	0.0028 (9)
C3	0.0307 (10)	0.0235 (10)	0.0261 (11)	0.000	0.000	−0.0014 (9)

### Geometric parameters (Å, °)

**Table:**

Cu1—N2^i^	1.974 (2)	N2—C1	1.155 (3)
Cu1—N2	1.974 (2)	N3—C3	1.143 (3)
Cu1—N3^i^	2.021 (2)	C3—C2	1.457 (3)
Cu1—N3	2.021 (2)	C2—H2A	0.9800
N1—C1^ii^	1.312 (3)	C2—H2B	0.9800
N1—C1	1.312 (3)		
			
N2^i^—Cu1—N2	110.34 (11)	C1—N2—Cu1	172.85 (18)
N2^i^—Cu1—N3^i^	111.79 (4)	C3—N3—Cu1	166.44 (18)
N2—Cu1—N3^i^	111.79 (4)	N2—C1—N1	176.2 (2)
N2^i^—Cu1—N3	111.79 (4)	C3—C2—H2A	109.5
N2—Cu1—N3	111.79 (4)	C3—C2—H2B	109.5
N3^i^—Cu1—N3	98.91 (11)	H2A—C2—H2B	109.6
C1^ii^—N1—C1	117.0 (3)	N3—C3—C2	179.6 (2)
			
N2^i^—Cu1—N3—C3	117.87 (5)	N3^i^—Cu1—N3—C3	0.000 (4)
N2—Cu1—N3—C3	−117.87 (5)		

Symmetry codes: (i) −*x*+3/2, −*y*+3/2, *z*; (ii) −*x*+1/2, −*y*+3/2, *z*.
